# Calculation of Radiative Properties for [82%Ar-18%CO_2_]-Fe Plasmas in MAG Welding Arc

**DOI:** 10.3390/ma15186415

**Published:** 2022-09-15

**Authors:** Fei Wang, Hongbing Liu, Xiaoli Liu, Lingfeng Zhang, Po Yang, Tianli Zhang, Zhishui Yu, Huan Li, Yann Cressault

**Affiliations:** 1 School of Materials Engineering, Shanghai University of Engineering Science, Shanghai 201620, China; 2 Shanghai Collaborative Innovation Center of Laser Advanced Manufacturing Technology, Shanghai 201620, China; 3 Hebei Special Equipment Supervision and Inspection Institute, Shijiazhuang 050200, China; 4 Tianjin Key Laboratory of Advanced Joining Technology, Tianjin University, Tianjin 300072, China; 5 Laboratoire Plasma et Conversion d’Energie (LAPLACE), Université de Toulouse, UPS, INPT, CEDEX 09, F-31062 Toulouse, France

**Keywords:** radiative properties, Ar-CO_2_-Fe plasmas, metal active arc welding, molecular emission, metal vapor

## Abstract

This paper is dedicated to the calculation of the radiative properties of 82%argon-18%CO_2_ thermal plasmas with the addition of metallic vapors (iron, in the present case, due to workpiece and wire erosion), this mixture being representative of metal active gas (MAG) arc welding processes. These radiative properties are obtained in the frame of the net emission coefficient (NEC) theory, using the recent and accurate “line by line” method. All significant radiative contribution mechanisms are taken into account in the calculation: atomic lines, atomic continuum (radiative attachment, radiative recombination, and bremsstrahlung), molecular bands for diatomic and polyatomic molecules, and molecular continuum. Broadening phenomena (Doppler and pressure effects) are also carefully treated for bound-bound transitions (atomic lines and molecular bands). Regarding 82%Ar-18%CO_2_ plasma, the results obtained demonstrate the key role of molecular bands at low temperatures (*T* < 4 kK), whereas the atomic line and continuum prevailed at intermediate and high temperatures. With the addition of a few percentages of iron vapor, it was shown that the total NEC is significantly increased (especially at low temperatures) and that the atomic and ionic lines become dominant in all the studied temperature ranges (3–30 kK). This theoretical study will constitute a groundwork to build a diagnostic method (based on the calculation of partial NECs for accurately chosen spectral intervals) for the determination of plasma temperature and iron vapor concentration in welding arcs.

## 1. Introduction

Gas metal arc welding (GMAW) is an efficient tool for joining metals in many industrial fields, e.g., in the construction of buildings, vehicles, ships, pipelines, and pressure vessels. GMAW is also referred to as metal inert gas (MIG) welding or metal active gas (MAG) welding, depending on whether the shielding gases are inert (e.g., Ar or He) or active (containing CO_2_ or O_2_). An Ar-CO_2_ mixture is the most popular shielding gas used in MAG welding for joining unalloyed steels, due to its low cost and good performance in arc stability and wetting power [1]. In the GMAW process, an arc is utilized to melt the workpiece to form the weld pool and melt the wire into droplets; metal vapor is inevitably produced on the molten metals due to the large thermal fluxes from the arc.

According to Murphy’s review [2], the presence of metal vapor has a major influence on arc welding. It can markedly increase the radiative emission [3] and the electrical conductivity [4] at low temperatures with small proportions (<1% by mole). It also can change other plasma properties, such as the viscosity or thermal conductivity, with proportions more than 20% by mole [4,5]. All of these effects result in changes in the a we current and energy transfer to the workpiece and thus, the profile of the weld pool [6,7,8]. Hence, it is essential to determine the metal vapor concentration for understanding the arc welding process. In addition to metal vapor concentration, temperature is another key parameter for plasmas. The plasma properties in GMAW arcs, such as transport and radiative properties, can be expressed as a function of these two parameters (e.g., [3,4]).

Although there exist numerous experimental methods, such as Thomson scattering [9,10,11] or Langmuir probe [12,13] to determine arc properties, optical emission spectroscopy (OES) has been by far the most commonly used method for diagnosing welding arcs. It has been widely used to measure some steady arcs, e.g., the arc of gas tungsten arc welding [14,15,16,17]. However, there are still many difficulties for GMAW arcs, because their arcs are highly dynamic due to metallic transfer and because the radiative properties used for plasma diagnosis are not always available. Therefore, few researchers have addressed the measurement of temperature and metal vapor concentration for GMAW arcs [18,19,20,21,22,23,24,25]. 

There is a consensus on the temperature measured: the temperature drops in the center of arcs in argon dominant shielding gases [18,19,20,21,22,23,24,25], but the variation in metal vapor concentration seems to be surprisingly large despite different welding conditions used. Goecke et al. [25] found a maximum concentration of 30%, Rouffet et al. [18] up to 60%, while Valensi et al. [19] measured less than 1% iron concentration. Moreover, the previous methods, which are based on the principle of line emission and the instruments of spectroscopy or intensified charge-coupled device (ICCD), can only instantaneously record the data for one spatial position or one layer of the arc. Hence, it is hard to diagnose the entire arc. In addition, most previous works are focused on MIG arcs [18,19,24,25], but few works are devoted to MAG arcs in an Ar-CO_2_ mixture [20,22]. As a consequence, there remains a need for an alternative approach that can precisely and quickly determine the temperature and the metal vapor concentration for GMAW arcs, especially for the MAG arc.

A method using a high-speed camera coupled with narrow bandpass filters may be an alternative to answer these requirements. A camera with many imaging units serves as multiple spectroscopies and quickly collects the integrated radiation within a spectral interval for the entire arc. The parameter, e.g., temperature, can be deduced by comparing the measured absolute or relative local emission with the theoretical emission. This method has been tested and validated in measuring the temperature for some plasmas with fixed components, e.g., pure Ar [26,27], Ar-H2, and Ar-He-H_2_ [28] in the case of a stabilized torch or air [29], in steady or unsteady conditions. However, it is difficult to find the determination of both the temperature and the component concentration for plasmas with an unfixed component. 

This study is part of research concerning the development of the high-speed camera method that allows the determination of the temperature and the metal vapor concentration in MAG arcs with a solid steel wire (Ar-CO_2_-Fe plasmas). The most critical challenge of this method is to select the appropriate diagnostic spectral intervals, which requires the prerequisite knowledge of the radiative properties of plasmas [29]. Hence, this paper is devoted to the theoretical study of the radiative properties of Ar-CO_2_-Fe plasmas. Based on the results, it will be easy to diagnose MAG arcs by selecting suitable spectral intervals. In this study, the radiation spectra of [82%Ar-18%CO_2_]-Fe plasmas with various iron content were calculated, and their integrated radiation was analyzed by the net emission coefficient method (NEC) to facilitate the selection of spectral intervals. The shielding gas of 82% Ar-18% CO_2_, typically, e.g., Linde Industrial Gases CORGO 18, is adopted due to its wide use in MAG welding [1]. Considering the boiling point of iron (3032 K) and the operating pressure of GMAW (at atmosphere pressure), we chose the lowest calculation temperature as 3 kK and the pressure as 0.1 MPa.

It should be noted that the radiative properties have already been reported for pure gases, binary gas mixtures, or more complex mixtures containing argon, carbon dioxide, or iron: Ar [3], CO_2_ [30], Fe [31], Ar-Fe [3], CO_2_-Cu [32], Ar-H_2_-Fe [33], and CO_2_-N_2_-Cu [34] but to the best of our knowledge, there are no available data for Ar-CO_2_ or Ar-CO_2_-Fe plasmas. In addition, to obtain fine spectra, two special treatments were involved in the calculation: the “line by line” method and the consideration of molecular radiation. Indeed, the emission of atomic lines is usually considered through the escape factor [35] by neglecting the overlapping of lines which spares calculation time but tends to overestimate the NEC [5]. Due to numerous iron lines (94,920 lines) in plasmas, we considered line overlapping by using the “line by line method” [36,37]. In addition, when the temperature is lower than 10 kK, the contribution of molecular species should not be neglected in presence of oxygen [38] and carbon [39]. Because the large area of GMAW arcs is below 10 kK [18,19,20,21,22,23,24,25], we considered the emission of the molecular systems of O_2_, CO, CO^+^, C_2_, CO_2,_ and O_3_.

## 2. Plasma Composition

Because the radiative properties of plasmas depend strongly on the chemical species in the mixture, we first calculated the equilibrium composition for the Ar-CO_2_-Fe plasmas with different iron content in the temperature range of 3 kK–30 kK and at atmospheric pressure. The molar ratio between Ar and CO_2_ is constant at 82%/18% due to the use of an 82% Ar-18% CO_2_ mixture. In this study, the calculation of plasma composition was realized by solving the coupled equations that describe the mass action law and the conservation of atomic nucleus, electrical neutrality, and particle density (pressure conservation). To achieve a fast solution, we adopted the method proposed by Godin and Trepanier [40] which is based on the chemical base concept. In addition, the pressure corrections were also taken into account: Virial correction at low temperature [41] and Debye–Hückel correction at high temperature [42]. For detailed calculations, please refer to our previous paper [43]. Table 1 lists the 39 chemical species considered in this work.

Internal partition functions (IPF) are essential data for the calculation of plasma composition. IPFs of atoms and their positive ions were calculated with the degeneracies and electronic level energies taken from the NIST database [44]; these IPFs were systematically compared with Drawin and Felenbok’s IPF complication [45]. For the negative ions, IFP was assumed to be equal to the degeneracy of the ground state. According to Herzberg [46], we adopted the Morse potential minimization method [47] to calculate the IPF of the diatomic molecules. For polyatomic molecules, IPF was calculated according to Herzberg [48] in the frame of the harmonic oscillator and the rigid rotator assumptions. The required spectroscopic data (Dunham coefficients, moments of inertia, degeneracies, vibrational frequencies, and symmetry number) were taken from Huber and Herzberg [49] and Chase et al. [50]. 

**Figure 1:**
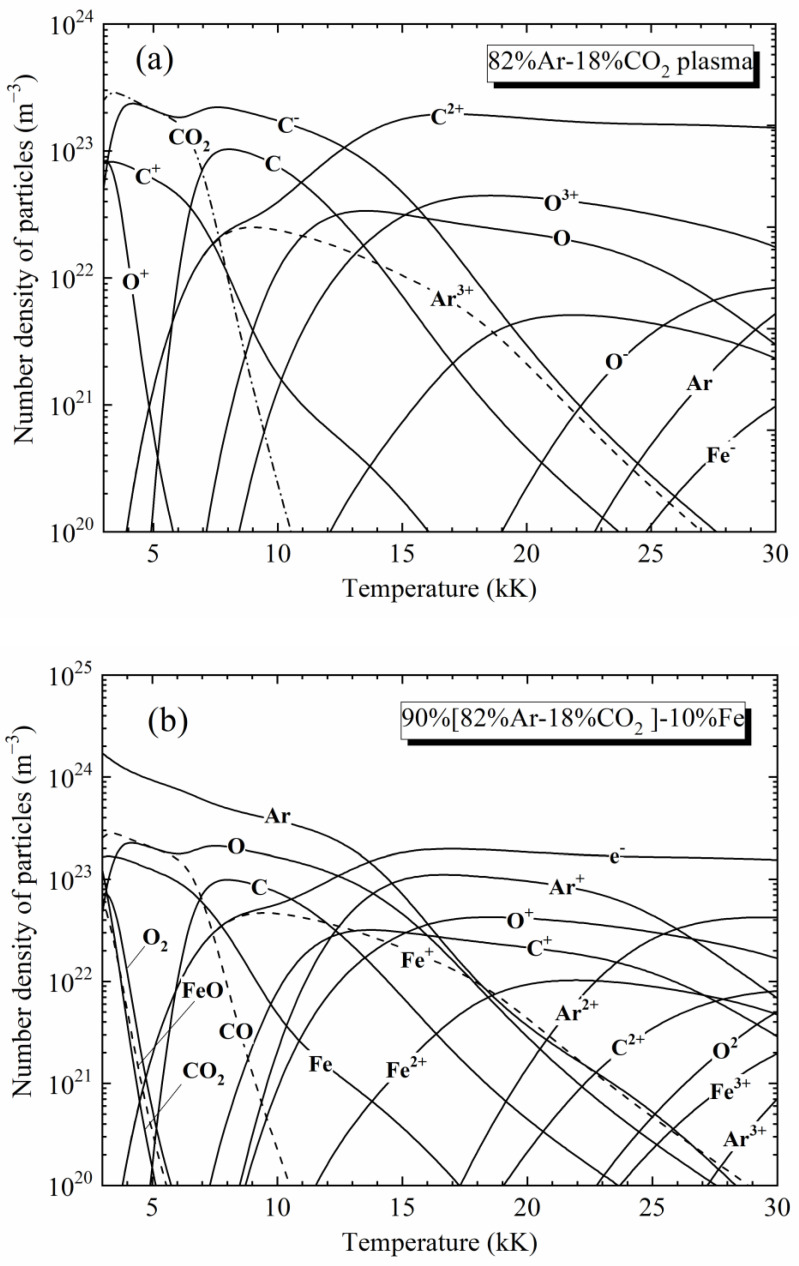

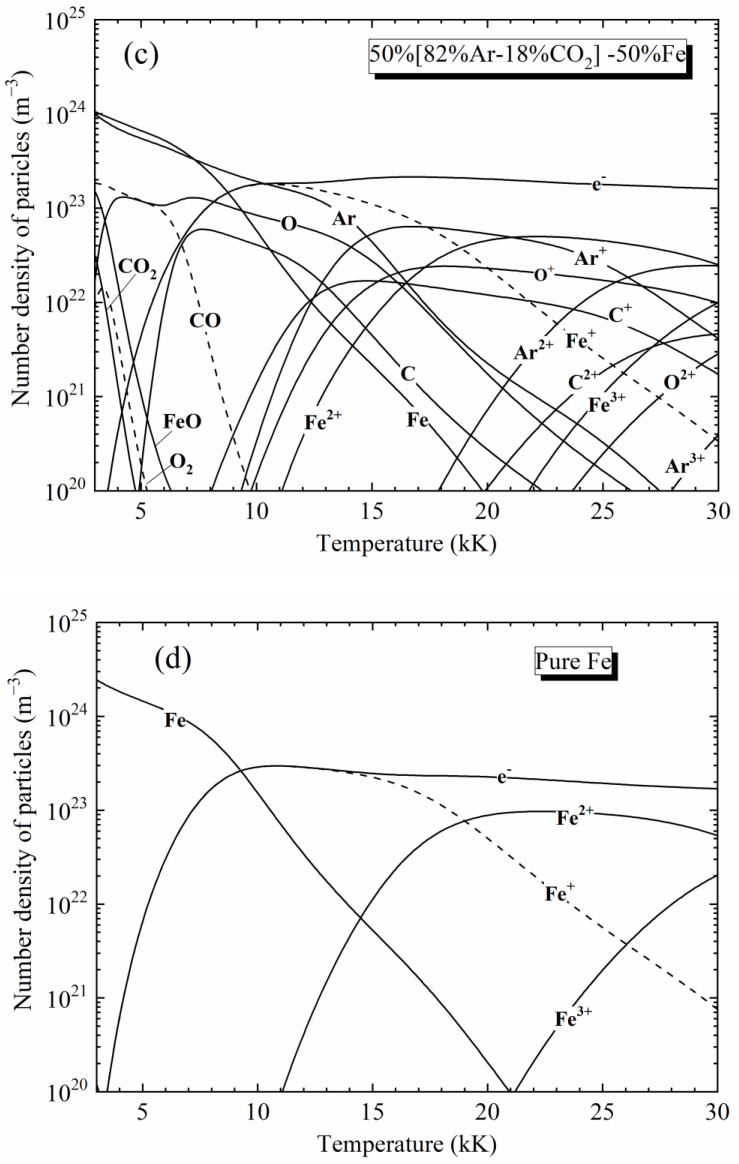
Equilibrium composition of some [82%Ar-18%CO_2_]-Fe mixtures (molar proportion) at 0.1 MPa: (**a**) 82%Ar-18%CO_2_; (**b**) 90%[82%Ar-18%CO_2_]-10%Fe; (**c**) 50%[82%Ar-18%CO_2_]-50%Fe; and (**d**) Pure Fe.

Figure 1 shows the compositions of some [Ar-CO_2_]-Fe mixtures at 0.1 MPa, using the 90%[82%Ar-18%CO_2_]-10%Fe mixture, as an example. At low temperatures (*T* < 4 kK), the plasma is dominated by the neutral atoms (Ar, Fe, O) and the molecules (CO_2_, CO, O_2,_ and FeO). Above 4 kK, the Fe^+^ ions gradually increase, and above 8 kK, the ions C^+^, Ar^+^, and O^+^ successively appear and increase together with the electrons; this rise of the ion population is due to the lower ionization energy of iron (7.902 eV) compared with those of others (11.260 eV, 13.618 eV, and 15.760 eV for C, O, and Ar, respectively). Above 15 kK, plasma is mainly composed of charged particles.

## 3. Radiative Properties

### 3.1. Radiative Mechanisms

#### 3.1.1. Radiation from the Atomic Lines

The line emission results from the spontaneous transition of an excited electron from a high energy level E_i_ to a lower energy E_j_. In the calculation, 117,213 atomic lines (6672 lines for atomic carbon species, 6217 lines for atomic oxygen species, 9404 lines for atomic argon species, and 94,920 lines for atomic iron species) are taken into account. The monochromatic emission coefficient is expressed as [3]:

(1) $$\varepsilon_{\lambda }^{\mathrm{lines}}\left(T\right)=\frac{\mathrm{hc}}{{4\pi \lambda }_{\mathrm{ij}}}\cdot A_{\mathrm{ij}}\cdot N_{i}\left(T\right)\cdot P_{\mathrm{ij}}^{\mathrm{Voigt}}\left(\lambda \right)$$

where *λ* is the wavelength, *h* and *c* are the Planck constant and the speed of light, respectively, *λ_ij_* is the transition wavelength, *A_ij_* is the spontaneous emission probability issued from the table of Moore [51], NIST table [44], and Kurucz and Peytremann [52], *N_i_* is the population number density of the particles in their excited states, and *P_ij_*(*λ*) is the normalized Voigt profile of the line.

Usually, in order to simplify the treatment of the spectra, the line overlapping is neglected, each line is treated separately through the escape factor proposed by Drawin and Emard [35]. This factor is defined for a line as the ratio of the radiative flux escaping from isothermal plasma with thickness *R*_p_ to the radiative flux escaping from optically thin plasma. Unfortunately, this treatment tends to overestimate the NEC. The final objective of this global work is the determination of the plasma temperature based on a fine description of the spectrum, we chose to calculate the monochromatic emission coefficient of a line according to the “line by line” method [36,37] since the escape factor does not allow a fine spectral description of the lines. Assuming this approach, the main difficulty of the calculation is the determination of the line’s profiles. In this work, we took into account the broadening phenomena resulting from Doppler and pressure effects (Van der Waals broadenings, resonance broadenings, and Stark broadenings). The line shape of Doppler broadening is assimilated to a Gaussian profile, and the full width at half maximum (FWHM) is given by [53,54]. The pressure broadenings, whose line shape is described according to a Lorentzian profile, are caused by the interaction of an emitting atom with surrounding particles. The FWHMs of resonance broadenings and Van de Waals broadenings are given by [55,56], and Stark broadenings are given by [54,57]. The convolution of the Gaussian and the Lorenzian functions results in a Voigt profile, whose analytical form is given by Whiting [58], as follows:

(2) $$P_{\mathrm{ij}}^{\mathrm{Voigt}}\left(\lambda \right)=\frac{2\ln2}{{\delta \lambda }_{D}^{2}}\cdot \frac{{\delta \lambda }_{L}}{\pi^{3/2}}\int_{-\infty }^{+\infty }\frac{\exp\left({-x}^{2}\right)}{{\left(\sqrt{\ln2}\;\frac{{\delta \lambda }_{L}}{{\delta \lambda }_{D}}\right)}^{2}+{\left(x\;-\;y\right)}^{2}}\cdot dx$$

$$\mathrm{with}\;y=\frac{2\;\ln2}{{\delta \lambda }_{D}^{2}}\cdot \left({\lambda \;-\;\lambda }_{\mathrm{ij}}\right)$$

with $\delta \lambda_{D}$ and $\delta \lambda_{L}$ being the Doppler and the Lorentz broadenings, respectively.

#### 3.1.2. Radiation from the Atomic Continuum

The radiation coming from the atomic continuum is produced by three mechanisms [3]:

- Radiative attachment: even if the radiative attachment is often negligible, we nevertheless considered this mechanism due to the presence of negatively charged particles (C^−^, O^−^, Fe^−^) in the plasma mixtures. Knowing the electronic affinity for the different species (C = 121.9 kJ/mol, O = 141 kJ/mol, and Fe = 15 kJ/mol), the monochromatic emission coefficient was calculated according to [3]:
  (3) $$\varepsilon_{\lambda }^{\mathrm{att}}\left(T\right)=\left(\frac{2\mathrm{hc}}{\lambda^{3}}\right)\cdot \left(\frac{c}{\lambda^{2}}\right)\cdot \exp\left(-\frac{\mathrm{hc}}{{\lambda k}_{B}T}\right)\cdot n_{A}-\left(T\right)\cdot \sigma_{\det}\left(\lambda \right)$$
  where k_B_ is the Boltzmann constant, $n_{A}-\left(T\right)$ is the population number density of the negatively charged particles, and $\sigma_{\det}\left(\lambda \right)$ is the photo-detachment cross-section taken from Yachkov for carbon [59], and Robinson and Geltman for oxygen [60]. We did not find data to take into account the attachment of iron.
- Radiative recombination: radiative recombination occurs when an electron and an atomic ion can recombine. This mechanism is often important in the continuum radiation of thermal plasmas. Its calculation requires the knowledge of the Biberman–Schluter factors, which are performed by summing the photoionization cross sections for all the considered energy levels, assuming a Thomas–Fermi shielded potential [61]. For carbon, oxygen, and argon species, this factor has been calculated and tabulated by Hofsaess [62]. The corresponding monochromatic emission coefficient is defined by [3,63]:
  (4) $$\varepsilon_{\lambda }^{\mathrm{rec}}\left(T\right){=C}_{1}\cdot \left(\frac{c}{\lambda^{2}}\right)\cdot \frac{n_{e}\left(T\right)\cdot n_{z+}\left(T\right)}{Q_{z+}^{\mathrm{int}}\left(T\right)}\cdot \frac{Z_{z+}^{2}}{\sqrt{T}}\cdot \left[1\;-\;\exp\left(-\frac{\mathrm{hc}}{{\lambda k}_{B}T}\right)\right]\cdot g_{1}^{z+}\cdot \xi_{\lambda }^{\left(z-1\right)}\left(T\right)$$
  This is example 1 of an equation:
  $$\mathrm{With}\;C_{1}=\frac{16\pi {\left(e^{2}/{4\pi \varepsilon }_{0}\right)}^{3}}{{3c}^{3}\sqrt{{6k}_{B}{\pi m}_{e}^{3}}}=5.44436\;\times \;10^{-52}\;J\;m^{3}\;K^{1/2}\;{\mathrm{sr}}^{-1}$$
  where $n_{e}\left(T\right)$ and $n_{z+}\left(T\right)$ are the population number densities of electron and ions, respectively, and $Z_{z+}$ is the charge of the ion *A^z^*^+^. $Q_{z+}^{\mathrm{int}}\left(T\right)$ and $g_{1}^{z+}$ are the internal partition function and the ground level degeneracy of the ion *A^z^*^+^, respectively, *m*_e_ and *e* are the mass and the charge of the electron, respectively, $\varepsilon_{0}$ is the permittivity of vacuum, and $\xi_{\lambda }^{\left(z-1\right)}$ is the Biberman–Schluter factor issued from Hofsaess [62,64] for argon (Ar and Ar^+^), carbon (C and C^+^), and oxygen (O, O^+^, and O^2+^). As the Biberman–Schluter factor was not available for iron species and multi-charged species (Ar^2+^, Ar^3+^, C^2+^, C^3+^, and O^3+^), we used the hydrogen-like atoms approximation developed by Okuda et al. [65].
- Bremsstrahlung: the bremsstrahlung radiation is produced by the deceleration of an electron due to the deflection by an electric field. The monochromatic emission coefficients for electron-ion and electron-atom interactions are given, respectively, by
  (5) $$\varepsilon_{\lambda }^{\mathrm{ei},z+}\left(T\right){=C}_{1}\cdot \left(\frac{c}{\lambda^{2}}\right)\cdot Z_{z+}^{2}\frac{n_{e}\left(T\right)\cdot n_{z+}\left(T\right)}{\sqrt{T}}\cdot \exp\left(-\frac{\mathrm{hc}}{{\lambda k}_{B}T}\right)\cdot G_{\mathrm{ei},\;\lambda }^{z+}\left(T\right)$$
  (6) $$\varepsilon_{\lambda }^{\mathrm{ea}}\left(T\right){=C}_{2}\cdot \left(\frac{c}{\lambda^{2}}\right)\cdot n_{a}\left(T\right)\cdot n_{e}\left(T\right)\cdot T^{\;3/2}\cdot \exp\left(-\frac{\mathrm{hc}}{{\lambda k}_{B}T}\right)\cdot G_{\mathrm{ea},\;\lambda }\left(T\right)$$
  $$\mathrm{With}\;C_{2}=\frac{32}{{3c}^{3}}\cdot \left(\frac{e^{2}}{{4\pi \varepsilon }_{0}}\right)\cdot {\left(\frac{k_{B}}{{2\pi m}_{e}}\right)}^{3/2}=3.4218\;\times \;10^{-43}{\;J\;m\;K}^{-3/2}{\mathrm{sr}}^{-1}$$
  $n_{a}\left(T\right)$ is the neutral atom number density. For electron-ions interactions, the Gaunt factor $G_{\mathrm{ei},\;\lambda }^{z+}\left(T\right)$ is introduced to correct non-classical behavior using hydrogen-like approximation and is issued from the table calculated by Grant [66,67]. For electron-atoms interactions, the factor $G_{\mathrm{ea},\;\lambda }\left(T\right)$ is homogeneous to a surface and depends on the elastic cross-section which is taken from Neynaber et al. [68] and Robinson and Geltman [60] for carbon and oxygen, and from Tanaka and Lowke [69] for argon.

#### 3.1.3. Radiation from the Atomic Continuum

The molecular continuum can have an important impact on the surrounding regions where the radiation coming from the hottest regions can be strongly absorbed, especially the UV radiations. Consequently, we included this phenomenon in the calculation assuming its importance for temperatures lower than 10 kK. To take into account this radiative process, we have to consider the photodissociation corresponding to the dissociation of a molecule by photon absorption (AB+hν⇄ A+B), the simple photoionization corresponding to an electron loss by photon absorption $\left(\mathrm{AB}+h\nu ⇄{\;\mathrm{AB}}^{+}{+e}_{\;}^{-}\right)$, and the dissociative photoionization corresponding to the dissociation of a molecule ($\mathrm{AB}+h\nu \;⇄{\;A+B}^{+}{+e}_{\;}^{-}$). As it is very difficult to calculate the cross-sections for all the rotational, vibrational, or electronic levels, we preferred to use experimental data from the literature. Nevertheless, these data are often obtained in the Standard Temperature Conditions at atmospheric pressure. Based on this report, we applied two assumptions: (1) we only considered the fundamental energy levels, which is a good approximation for the low temperatures where the population number densities of the molecules are significant; (2) the cross-sections of the various involved radiative mechanisms were supposed to be independent of the temperature and only dependent on the wavelength:

(7) $$\varepsilon_{\lambda }^{\mathrm{MC}}\left(T\right){=B}_{\lambda }\left(T\right)\cdot N_{A_{2}}\left(T\right)\cdot \sum_{i}\sigma_{A_{2}}\left(\lambda ,300\;K\right)\cdot \left(1\;-\;\exp\left(-\frac{\mathrm{hc}}{{\lambda k}_{B}T}\right)\right)$$

where $\varepsilon_{\lambda }^{\mathrm{MC}}\left(T\right)$ is the corresponding spectral emission,${\;B}_{\lambda }\left(T\right)$ is the Planck function, $N_{A_{2}}\left(T\right)$ the total number density of the molecule *A*_2_ at the temperature *T* (in m^−3^), and $\sigma_{A_{2}}$ is the total photoabsorption cross-section of the same molecule (in m^2^). In this work, we considered the molecules C_2_, O_2_, CO, CO_2_, and O_3_ already studied in the works of Jan et al. [70] and Billoux et al. [30]. All the references used for the photoabsorption cross-sections are reported in Table 2.

#### 3.1.4. Radiation of the Molecular Bands

The consideration of the molecular bands in the radiative spectra is an important task of this work since it was rarely included in our previous works [5], except in the last works of Billoux et al. [30,32]. Here, we took into consideration the diatomic molecular systems of O_2_, CO, CO^+^, and C_2_ and the polyatomic molecular systems of CO_2_ and O_3_, even if some of them have a low contribution rate in the plasma composition such as CO_+_ or O_3_, as listed in Table 3. They have been validated in Air and CO_2_ by Babou et al. (for CO, CO_+_, C_2_, O_2_, and CO_2_) [83], Chauveau et al. (for O_2_, CO, and C_2_) [84], Lino Da Silva and Dudeck (for CO) [85], and Laux (for O_2_) [53].

- *Diatomic molecular systems:* the emission coefficient of each molecular line between two rotational levels $J^{\prime }$ and $J^{\prime \prime }$ is given by [33]:
  (8) $$\varepsilon_{\lambda }^{\mathrm{MB}}\left(T\right)=\frac{\mathrm{hc}\sigma }{4\pi }\cdot N_{A_{2}}\left(n^{\prime },\;\nu^{\prime },\;K^{\prime },\;J^{\prime },\;P^{\prime }\right)\cdot A_{n^{\prime \prime }\;,\;\nu^{\prime \prime },\;K^{\prime \prime },\;J^{\prime \prime }}^{n^{\prime },\;\nu^{\prime },\;K^{\prime },\;J^{\prime }}$$
  where *σ* is the wavenumber, $N_{A_{2}}\left(n^{\prime },\;\nu^{\prime },\;K^{\prime },\;J^{\prime },\;P^{\prime }\right)$ is the population number density of the emitting level, and $A_{n^{\prime \prime },\;\nu^{\prime \prime },\;K^{\prime \prime },\;J^{\prime \prime }}^{n^{\prime },\;\nu^{\prime },\;K^{\prime },\;J^{\prime }}$ is the transition probability.

Assuming a Boltzmann distribution, the population number density of the emitting level is defined by:

(9) $$N_{A_{2}}\left(n^{\prime },\;\nu^{\prime },\;K^{\prime },\;J^{\prime },\;P^{\prime }\right)=\left(\frac{N_{A_{2}}\left(T\right)}{Q_{A_{2}}^{\mathrm{int}}\left(T\right)}\right)\phi \left({2J}^{\prime }+1\right)\exp\left(-\frac{E^{\prime }}{k_{B}T}\right)$$

(10) $$\mathrm{with}\;E^{\prime }=\mathrm{hc}\left(T_{e}^{\prime }+G^{\prime }\left(\nu^{\prime }\right)+F_{\nu }^{\prime }\left(K^{\prime }\right)\right)$$

where $N_{A_{2}}\left(T\right)$ is the number density of the molecule *A*_2_, $Q_{A_{2}}^{\mathrm{int}}\left(T\right)$ is the internal partition function of the molecule *A*_2_ depending on the parity of the level and the spin quantum number, $n^{\prime }$ is the electronic level, and $\nu^{\prime }$, $K^{\prime }$, $J^{\prime },$ and $P^{\prime }$ are the vibrational, rotational, sub-rotational, and symmetry quantum numbers, respectively; $E^{\prime }$ is the energy of the level in the Born-Oppenheimer approximation, and $T_{e}^{\prime }$, $G^{\prime }\left(\nu^{\prime }\right),$ and $F_{\nu }^{\prime }\left(K^{\prime }\right)$ are the electronic, vibrational, and rotational energies, respectively.

The transition probability of a radiative transfer is obtained by the expressions:

(11) $$A_{n^{\prime \prime },\;\nu^{\prime \prime },\;K^{\prime \prime },\;J^{\prime \prime }}^{n^{\prime },\;\nu^{\prime },\;K^{\prime },\;J^{\prime }}=A_{n^{\prime \prime },\;\nu^{\prime \prime }}^{n^{\prime },\;\nu^{\prime }}\;\cdot \;A_{K^{\prime \prime },\;J^{\prime \prime }}^{K^{\prime },\;J^{\prime }}$$

(12) $$\mathrm{with}\;A_{K^{\prime \prime },\;J^{\prime \prime }}^{K^{\prime },\;J^{\prime }}=\frac{S_{K^{\prime \prime },\;J^{\prime \prime }}^{K^{\prime },\;J^{\prime }}}{2J^{\prime }+1}$$

where $A_{n^{\prime \prime },\;\nu^{\prime \prime }}^{n^{\prime },\;\nu^{\prime }}$ is the Einstein coefficient, $A_{K^{\prime \prime },\;J^{\prime \prime }}^{K^{\prime },\;J^{\prime }}$ is the rotational transition probability, and $S_{K^{\prime \prime },\;J^{\prime \prime }}^{K^{\prime },\;J^{\prime }}$ is the Hönl-London factor calculated by Whiting et al. [86]:

(13) $$\sum S_{K^{\prime \prime },\;J^{\prime \prime }}^{K^{\prime },\;J^{\prime }}=\left({2-\delta }_{{0,\;\Lambda }^{\prime }}\delta_{{0,\;\Lambda }^{\prime \prime }}\right)\left(2S^{\prime }+1\right)\left(2J^{\prime }+1\right)$$

where $\delta_{0,\;\Lambda }$ is the Kronecker symbol, whose value is 1 if Λ=0 and 0 otherwise, $2S^{\prime }+1$ is the spin multiplicity of the upper electronic state. $\Lambda =\left|M_{L}\right|$ is the quantum number, with a value between 0 and  L (state $\;\Sigma \left(\Lambda =0\right)$, $\Pi \left(\Lambda =1\right)$, $\Delta \left(\Lambda =2\right)$).

Concerning the broadening phenomena of the diatomic lines, we took into consideration Doppler and collisional broadenings. For the Doppler effects, and in the case of molecules, we used the same formula as an atomic case. For collisional broadenings, it is different since we did not have enough information for all the temperature ranges. Therefore, we systematically used a semi-empirical approximation law to determine the molecular collisional broadenings with an HWHM (Half widths at Half Maximum) $\gamma_{L}$ as a function of the pressure, *P*. Depending on the wavelength region, we used the recommendation of Breene [87]:

(14) $$\gamma_{L}\left({\mathrm{cm}}^{-1}\right){=\gamma }_{0}\left(T_{\mathrm{ref}}\right)\cdot P\cdot {\left(\frac{T_{\mathrm{ref}}}{T}\right)}^{n}$$

The relation (14) has been validated by Sick et al. [88], Lewis et al. [89], and Chauveau [84] for O_2_ molecules. The work of Chauveau in air plasmas also indicated the low influence of this approximation on the radiative flux. The relation (14) can be applied because of the large broadenings of the molecular bands due to their high population number densities (leading to a less important role for the wings), because of the weak self-absorption of these molecular lines and because of the strong importance of the Doppler effect in most cases (excepted in the Infrared). Due to these last remarks, we decided to apply it to all the diatomic molecules. We can find that for visible and near IR (for O_2_ SR, for example), the values of *γ*_0_, Tref, and *n* are 0.105, 273, and 0.7, respectively [88,89]; in UV and molecules in Hitran, their values are 0.265, 295, and 0.66, respectively [90]; in IR, they are 0.053, 296, and 0.75, respectively [91].

- Polyatomic molecular systems: the radiation of the polyatomic species can be very important in the Infrared region. The radiation coming from CO_2_ was calculated using the database CDSD-4000 of Tachkun and Perevelov [92] which seems to be the most complete in the literature with wavenumbers between 8310 and 226 cm^−1^, and temperatures up to 4000 K. Similar to Billoux et al. [33], we did not consider the radiation of CO_2_ for higher temperatures due to a lower contribution to the radiative spectrum. For the O_3_ molecule, we used the HITRAN 2012 database [93], even if these molecules are in small concentrations in the plasma. The broadenings were described with a Voigt profile and collisional effects were defined by the relation (14). This last database gave us two kinds of broadenings: the broadenings parameters due to the perturbation of the species existing in air and the broadenings parameters corresponding to the perturbation of the molecular energy levels by species of the same type (self-broadenings). As this work is not focused on air plasmas, we considered all the broadening phenomena of a molecular line due to molecules of the same type with a total self-broadening. Under this consideration, we made two strong assumptions that we suppose not to be far from reality: (1) the molecular species is important in the plasma’s composition and the perturbation due to the other species can be neglected; (2) if the previous condition is not respected, all the perturbations caused by the other molecular species can be considered identically even if they are similar (or not) to the perturbed species. The second assumption can be debated but we supposed that low temperatures tend to increase the molecular species and reduce the impact on the broadening coefficients.

#### 3.1.5. Discretization and Integration of the Monochromatic Emission Coefficient (Equation (1))

The most rigorous method to describe the spectra is the “line by line” method in order to take into account the broadenings of the atomic and molecular lines. According to previous works using this method [31,39,70], we needed to find a compromise between a very fine description and a reduced computing time. Consequently, in this work, we calculated high-resolution spectra with a variable wavelength step, from 10^6^ to 7 × 10^6^ wavelengths, depending on the temperature (more wavelengths are needed at low temperatures to take into account the molecular bands). The lines were integrated over ±50 nm from the line center which is sufficient at atmospheric pressure [39,70] and led to 0.5% accuracy in this study compared to an integration on the total wavelength range. The algorithm of Drayson [94] was used to calculate the Voigt profile in order to reduce the computing time. 

### 3.2. Net Emission Coefficient

There are many methods to represent the spectral radiation from a given geometrical plasma, such as the NEC method [95], the Partial Characteristics method [96], the Discrete Ordinates Method [97], the k-distribution [98], the PN approximation [99], or the Monte Carlo method [100]. Some of these methods are detailed in the works of Siegel and Howell [101] and Modest [102]. However, the most popular method used in modeling and spectral diagnostics is the NEC method. The NEC method is defined as the divergence of the radiative flux at the center of a spherical, homogeneous, and isothermal plasma [95]. The NEC can be interesting for plasma diagnostics using a camera [29,30] since we can estimate the radiation of specific spectral intervals (we speak about “partial NEC”) and compare the results with measurements. Moreover, the NEC calculated over the total spectrum is widely accepted in modeling the hot zones of plasma [43,103,104]. However, the NEC is not useful for high-temperature gradients and in cold regions, more particularly, the edges of the plasma where absorption can be significant. The NEC is defined as follows [3]:

(15) $$\varepsilon_{N}\left({T,R}_{p}\right)=\int_{0}^{\infty }B_{\lambda }\left(T\right)\cdot K^{\prime }\left(\lambda ,\;T\right)\cdot \exp\left({-K}^{\prime }\left(\lambda ,\;T\right)\cdot R_{p}\right)\cdot d\lambda$$

where *R*_p_ is the plasma radius based upon the assumption of a plasma being spherical, homogeneous, and isothermal, $B_{\lambda }\left(T\right)$ is the Planck function, and $K^{\prime }\left(\lambda ,\;T\right)$ is the total monochromatic absorption coefficient which is correlated with the local emission coefficient by Kirchhoff’s law: $\varepsilon_{\lambda }\left(T\right){=B}_{\lambda }\left(T\right)K^{\prime }\left(\lambda ,\;T\right)$. The total monochromatic absorption coefficient $K^{\prime }\left(\lambda ,\;T\right)$ corrected by the induced emission is expressed as:

(16) $$K^{\prime }\left(\lambda ,\;T\right)=K\left(\lambda ,\;T\right)\cdot \left(1\;-\;\exp\left(-\frac{\mathrm{hc}}{{\lambda k}_{B}T}\right)\right)$$

where $K\left(\lambda ,\;T\right)$ is the total monochromatic absorption coefficient at the wavelength *λ* and the temperature *T* (without correction). The NECs are quoted for a given plasma radius $R_{p}$. This radius is chosen to be the radius of the high-temperature region of the arc where the absorption is mainly realized and it is about 1 mm for a welding arc [2]. 

## 4. Results

### 4.1. Spectra of [82%Ar-18%CO_2_]-Fe Plasmas

**Figure 2:**
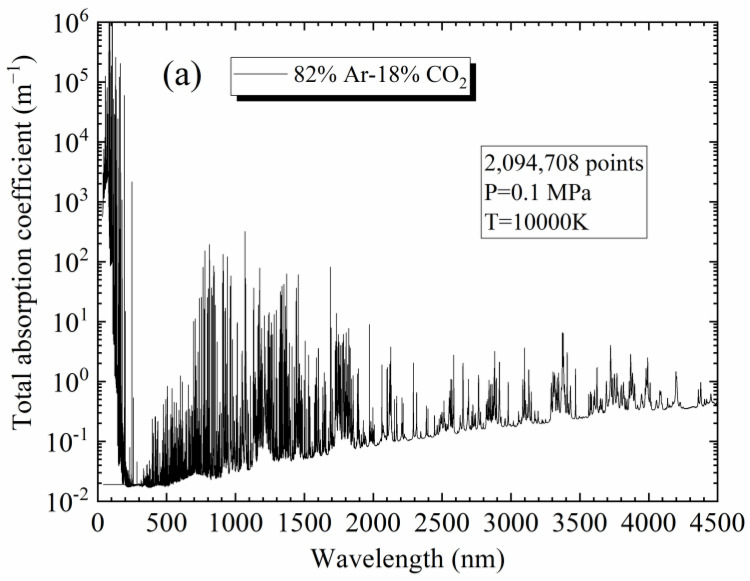

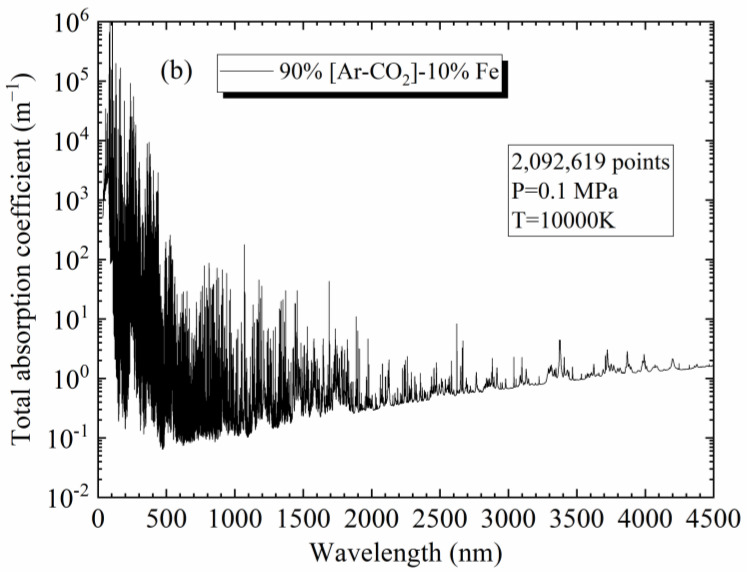
Total absorption coefficient at 0.1 MPa and 10,000 K for the thermal plasmas (**a**) 82%Ar-18%CO_2_; (**b**) 90%[82%Ar-18%CO_2_]-10%Fe (molar proportions).

In order to obtain the NEC (or “partial NEC”) for [82%Ar-18%CO_2_]-Fe plasmas, it is necessary to obtain their spectra prior to integrating them according to Equation (15). Figure 2a,b present the total absorption coefficient obtained at 10,000 K and 0.1 MPa for 82%Ar-18%CO_2_ and 90%[82%Ar-18%CO_2_]-10%Fe plasmas (molar proportions), respectively, as examples. For the 82%Ar-18%CO_2_ plasma, we can observe the presence of the molecular bands and molecular continuum at a low wavenumber, the contribution of a few lines at an intermediate wavenumber, and the role of the atomic continuum at a higher wavenumber. When 10%Fe is added to the plasma, more atomic lines appear in the spectrum due to the presence of numerous and emissive lines of iron, especially for low wavelengths.

### 4.2. Influence of Temperature and R_p_ on the NEC

**Figure 3:**
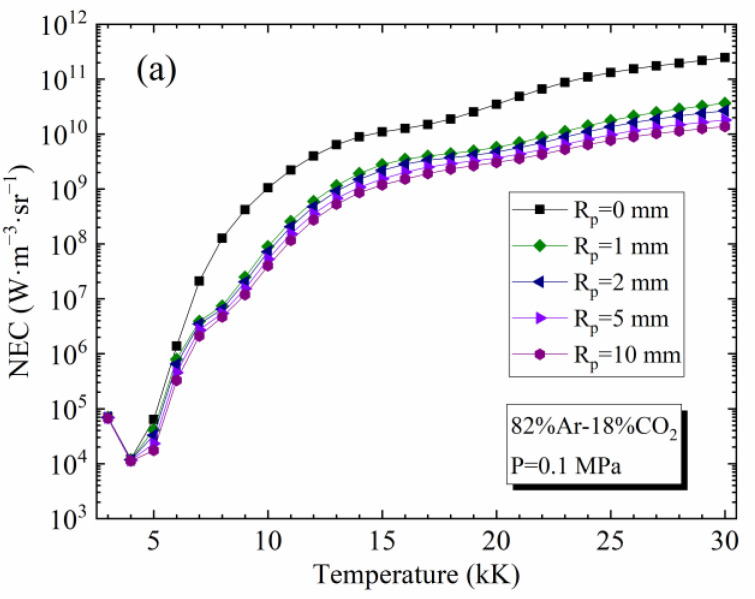

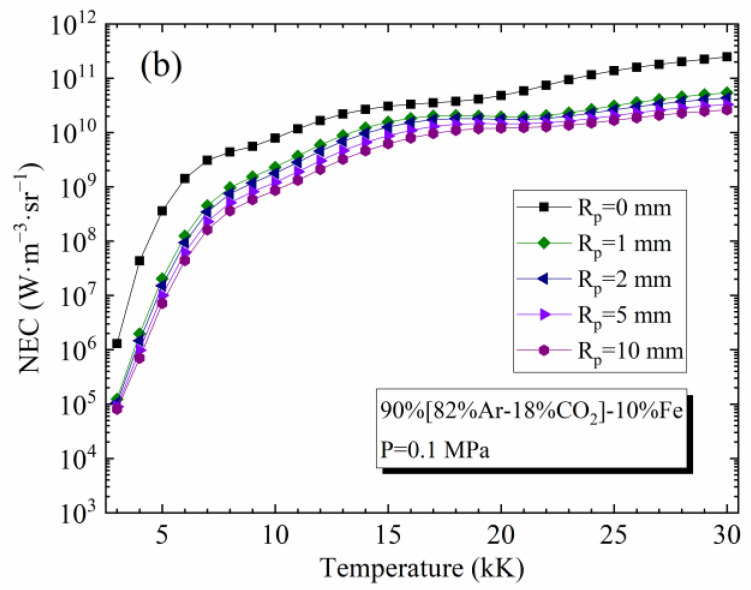
Influence of the plasma radius *R*_p_ on the NEC for the plasmas at atmospheric pressure: (**a**) 82%Ar-18%CO_2_ mixture; (**b**) 90%[82%Ar-18%CO_2_]-10%Fe (molar proportions).

Figure 3a,b presents NEC values for 82%Ar-18%CO_2_ and 90%[82%Ar-18%CO_2_]-10%Fe plasmas, respectively, at 0.1 MPa as a function of temperature and plasma radius *R*_p_. The case *R*_p_ = 0 mm corresponds to an optically thin plasma (without absorption). For 82%Ar-18%CO_2_ plasma, the NEC decreases with the temperature below 4 kK because the CO_2_ molecule is the main contributor to the radiation and its number density decreases rapidly due to dissociation. Above 4 kK, the NEC increases with the temperature because the radiation of CO begins to take effect at low temperatures, and the radiation of the atomic lines begins to take effect at higher temperatures. For 90%[82%Ar-18%CO_2_]-10%Fe plasma, since the stronger radiation of line emission is at low temperatures, the contribution of CO_2_ and CO radiation becomes secondary.

The NEC decreases with *R*_p_ due to the absorption phenomena and, more particularly, the absorption of the resonance lines of the atomic species (this phenomenon is more pronounced in presence of iron). The absorption is very important in the first millimeter. For the 82%Ar-18%CO_2_ plasma with a radius of *R*_p_ = 1 mm, 91.5% of the total absorption is achieved at 10 kK. Nevertheless, the absorption phenomenon is weak at very low temperatures because the plasma is dominated by the molecular species and their radiation is weakly absorbed. These conclusions are the same for other mixtures with and without metallic vapors.

### 4.3. Influence of Vapor Concentration on the NEC

**Figure 4:**
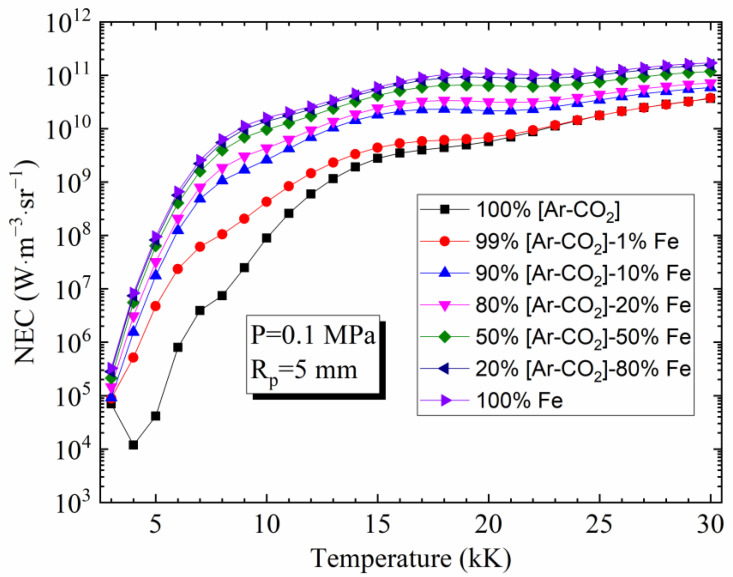
Influence of iron vapor on the NEC for [82%Ar-18%CO_2_]- Fe mixtures (molar proportions) at atmospheric pressure and *R*_p_ = 5 mm.

Figure 4 highlights the influence of vapor concentration on the NEC for *R*_p_ = 5 mm and [82%Ar-18%CO_2_]-Fe plasmas. As it can be seen, it is evident that the strong influence of iron on the NEC occurs with a very low concentration, especially at low temperatures. This is caused by two phenomena: the increase in the electron number density because of the low ionization potential of neutral iron compared to Ar, C, and O (7.902 eV, 15.760 eV, 11.260 eV, 13.618 eV for Fe, Ar, C, and O, respectively) and the rich spectrum of the atomic lines of neutral iron (more specifically, the resonance lines which are strongly emissive but also strongly absorbed). Similar behavior has also been observed for other plasmas with iron vapor, for example, an Ar-Fe mixture [3].

### 4.4. Influence of the Different Contributions to the Total Radiation

**Figure 5:**
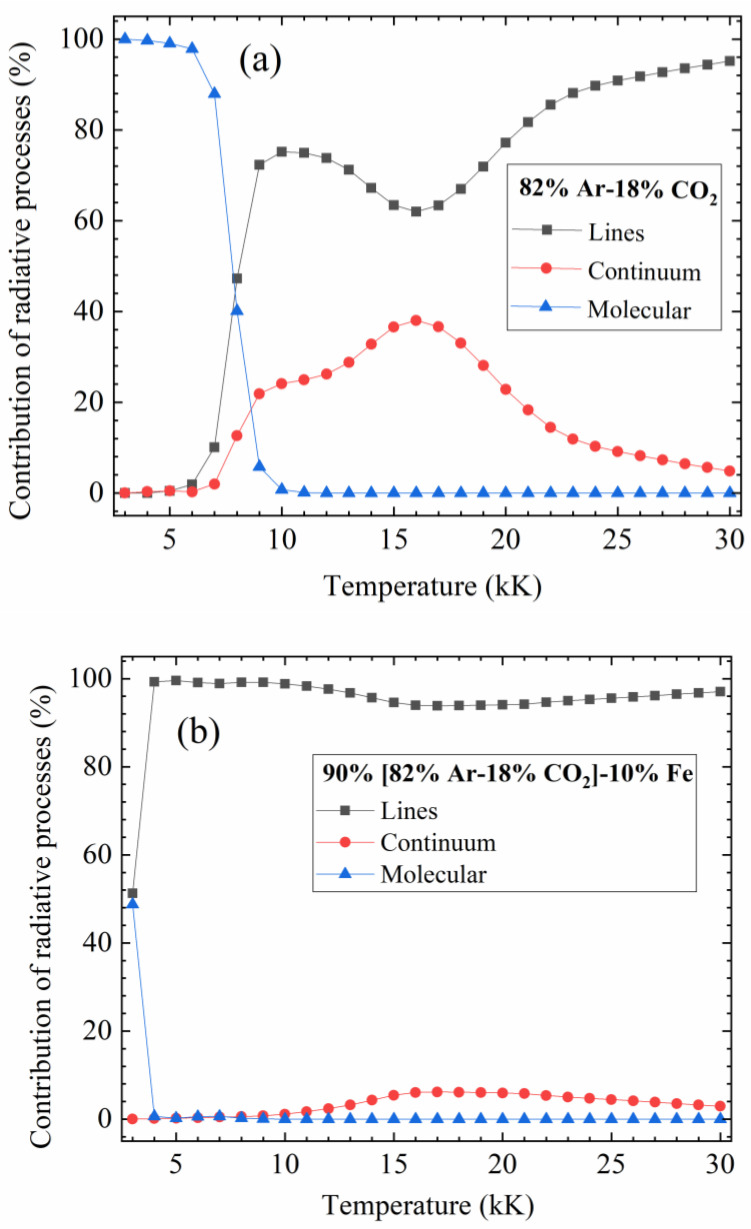
Contribution of the different radiative processes to the NEC for the plasmas at atmospheric pressure: (**a**) 82%Ar-18%CO_2_ mixture; (**b**) 90%[82%Ar-18%CO_2_]-10%Fe (molar proportions).

For a better understanding of the influence of various radiative mechanisms, the contributions of atomic lines, continuum (atomic continuum and molecular continuum), and molecular bands to the NEC are presented in Figure 5a,b for [82%Ar-18%CO_2_]-Fe plasmas without iron vapors and with an iron fraction of 10% (*R*_p_ = 5 mm, *P* = 0.1 MPa). In previous works of the Laplace laboratory (see [28]), we defined the spectral intervals for experiments by dividing the emission of the lines by the emission of the continuum in a specific spectral interval where the emission of the continuum did not vary significantly. Hence, it is important to know the variation of the different contributions to see the most important contributions as a function of the temperature range. It is also the occasion to validate our assumptions: the radiation of the molecules cannot be neglected at low temperatures for Ar-CO_2_ plasmas.

For the 82%Ar-18%CO_2_ plasma, we see that the radiation of molecular bands is the dominant contributor (>97%) to the NEC below 6 kK, and it shares the NEC with lines and continuum until 10 kK, with more than 50% at 7.5 kK. The radiation of the molecular bands is mainly produced by the CO_2_ system for temperatures below 4 kK, CO IR for temperatures between 3.5 kK and 5 kK, and 4th CO for higher temperatures. When temperatures exceed 10 kK, the radiation of the molecular bands becomes negligible, and the radiation of lines plays a leading role in the NEC while the continuum radiation also takes a considerable proportion, especially between 14 KK and 18 kK, accounting for above 30%.

When the plasma contains a low concentration of iron (e.g., 10% Fe in this case), it can be observed that the radiation of the atomic lines is the most important part (>90%) of the temperature range, and the other radiative mechanisms are of no significance. This is because the intense radiation of iron lines drastically minimizes the influence of molecular bands and continuum.

### 4.5. NECs for Certain Spectral Intervals

Because the NEC depends on the temperature *T* and the concentration of vapors *Y*_Fe_ for a given plasma size, it is possible to diagnose the *T* and *Y*_Fe_ in MAG arcs (with [82%Ar-18%CO_2_]-Fe plasmas) based on the NEC. Since the experimental instruments (CCD cameras) can only detect the radiation within certain spectral intervals (e.g., visible light), we actually need to find the NEC of spectral intervals (or “partial NEC”). By comparing the measured values with the theoretical ones, we can determine the *T* and *Y*_Fe_ in the arc.

**Figure 6:**
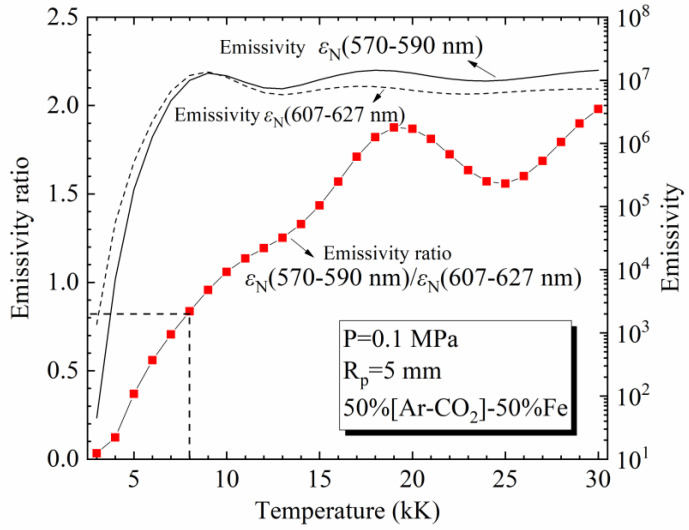
Net emission coefficients of the spectral intervals of 570–590 nm (line) and 607–627 nm (dash) and their emissivity ratio (line-square) at 0.1 MPa and *R*_p_ = 5 mm.

Let us consider the spectral intervals 570–590 nm and 607–627 nm as examples. Figure 6 shows their NECs and the ratio of these two partial NECs for a 50%[82%Ar-18%CO_2_]-50%Fe mixture at 0.1 MPa and *R*_p_ = 5 mm. For one position of the MAG arc, if the NEC (570–590 nm) and NEC (607–627 nm) measured are 1.00 × 10^6^ Wm^−3^sr^−1^ and 1.20 × 10^6^ Wm^−3^sr^−1^ (these values corresponding to the plasma with 50% Fe), respectively, we can observe in the figure that the ratio 0.836 corresponds to a temperature of 8 kK.

This method depends on the NECs of certain spectral intervals, and thus, the choice of the spectral intervals is a key issue. Since two parameters (*T* and *Y*_Fe_) are expected to be solved, at least two spectral intervals are needed. The NECs of ideal intervals should be sensitive to the variation of the temperature and iron concentration. In addition, the radiation in these spectral intervals must be weakly absorbed. This paper focuses on the calculation of the radiative properties of the [Ar-CO_2_]-Fe plasmas and demonstrates their radiative characteristics, while our companion paper will be devoted to finding two ideal spectral intervals ($\Delta \lambda_{1}$ and $\Delta \lambda_{2}$) and to diagnosing pulsed MAG arcs according to $\varepsilon_{\Delta \lambda_{1}}{(T,Y}_{\mathrm{Fe}})$ and $\varepsilon_{\Delta \lambda_{1}}{(T,Y}_{\mathrm{Fe}})/\varepsilon_{\Delta \lambda_{2}}{(T,Y}_{\mathrm{Fe}})$, in which the accuracy of our calculated spectra will be indirectly confirmed by comparison with the spectral measurement results [105].

## 5. Conclusions

This work is devoted to the determination of the radiative properties in the range 3–30 kK at 0.1 MPa for [82%Ar-18%CO_2_]-Fe plasmas representative of MAG arcs. The spectra and the NEC were obtained by considering all radiative mechanisms. It has been demonstrated that for the 82%Ar-18%CO_2_ plasma, the radiation of molecular bands is important at low temperature (*T* < 10 kK) and it is not self-absorbed in the plasma. At higher temperatures, atomic lines contribute the most to the radiation in spite of strong absorption, while the continuum radiation also assumes a considerable proportion. When a low iron concentration (e.g., 5%) exists in the plasma, the NEC is dramatically increased, especially at low temperatures. Atomic lines become the dominant contribution, thus making continuum and molecular bands insignificant.

This study mainly presents the entire spectra and corresponding NEC for [82%Ar-18%CO_2_]-Fe plasmas. It builds a foundation for the diagnostics of temperature and iron concentration in MAG arcs, which is based on the relation of the NEC of some particular spectral intervals and temperature and iron concentration, by using a high-speed CCD camera with narrow-band filters.

## Figures and Tables

**Table 1:** Chemical species at equilibrium state for the [82%Ar-18%CO_2_]-Fe plasmas.

Atoms, Atomic Ions, and Electron	Diatomic Molecules and Ions	Polyatomic Molecules and Ions
Ar, Ar^+^, Ar^2+^, Ar^3+^, C, C^−^, C^+^, C^2+^, C^3+^, O, O^−^, O^+^, O^2+^, O^3+^, Fe, Fe^−^, Fe^+^, Fe^2+^, Fe^3+^, and e^−^	C_2_, C_2_^+^, C_2_^−^, O_2_, O_2_^+^, O_2_^−^, CO, CO^+^, FeO, and Fe_2_	C_3_, C_3_^−^, CO_2_, CO_2_^−^, C_2_O, O_3_, C_4_, C_3_O_2_, and FeC_5_O_5_

**Table 2:** References used for photoabsorption cross-sections of major molecular species.

Molecule	References
C_2_	[71,72]
CO	[73,74,75]
O_3_	[76,77,78,79]
O_2_	[80]
CO_2_	[74,81,82]

**Table 3:** Molecular band systems taken into account in the present work.

Molecule	Electronic System	Electronic Transition	${(v}_{\max}^{\prime }{;v}_{\max}^{\prime \prime })$	$\sigma_{0,0}\left({\mathrm{cm}}^{-1}\right)$
O_2_	Schumann–Runge	$B^{3}\Sigma_{u}^{-}\to X^{3}\Sigma_{g}^{-}$	$\left(19;21\right)$	49,358
CO	Infrared	$X^{1}\Sigma_{\;}^{+}\to X^{1}\Sigma_{\;}^{+}$	$\left(49;40\right)$	-
	Fourth Positive	$A^{1}\Pi_{\;}\to X^{1}\Sigma_{\;}^{+}$	$\left(22;35\right)$	64,748
	Hopfield–Birge	$B^{1}\Sigma_{\;}^{+}{}_{\;}\to X^{1}\Sigma_{\;}^{+}$	$\left(2;50\right)$	86,916
	Angström	$B^{1}\Sigma_{\;}^{+}{}_{\;}\to A^{1}\Pi_{\;}$	$\left(2;20\right)$	22,171
	Third Positive	$b^{3}\Sigma_{\;}^{+}\to a^{3}\Pi_{\;}$	$\left(2;18\right)$	35,358
CO^+^	Comet-tail	$A^{2}\Pi_{\;}\to X^{2}\Sigma_{\;}^{+}$	$\left(30;26\right)$	20,408
	First negative	$B^{2}\Sigma_{\;}^{+}\to X^{2}\Sigma_{\;}^{+}$	$\left(30;35\right)$	45,633
	Baldet–Johnson	$B^{2}\Sigma_{\;}^{+}\to A^{2}\Pi_{\;}$	$\left(30;26\right)$	25,226
C_2_	Phillips	$A^{1}\Pi_{u}\to X^{1}\Sigma_{g}^{+}$	$\left(35;21\right)$	8268
	Mulliken	$D^{1}\Sigma_{u}^{+}\to X^{1}\Sigma_{g}^{+}$	$\left(22;21\right)$	43,668
	Deslandres–D’az.	$C^{1}\Pi_{g}\to A^{1}\Pi_{u}$	$\left(9;32\right)$	25,969

## Data Availability

Not applicable.
